# Biomechanical comparison of the tensile strength of fixation implants used for pull-out repair of medial meniscus posterior root tear

**DOI:** 10.1051/sicotj/2024034

**Published:** 2024-10-08

**Authors:** Mikiko Handa, Tsuneari Takahashi, Katsushi Takeshita

**Affiliations:** 1 Department of Orthopedic Surgery, Jichi Medical University 3311-1 Yakushiji Shimotsuke 329-0498 Japan; 2 Department of Orthopedic Surgery, Ishibashi General Hospital 1-15-4 Shimokoyama Shimotsuke 329-0596 Japan

**Keywords:** Meniscus, Root, Repair, Biomechanical study

## Abstract

*Purpose*: Medial meniscus posterior root tears (MMPRT) pull-out repair aims to restore the meniscus’ anatomical structure. Different implants are utilized for thread fixation in the pull-out repair technique for MMPRT. However, biomechanical evidence comparing the fixation strengths of these implants remains unavailable. This study investigated the tensile strength of three fixation implants in porcine knee models of MMPRT pull-out repair. *Methods*: This study categorized 30 porcine MMPRT models undergoing pull-out repair into three groups (10 specimens each) based on the implant utilized for fixation, including double spike plate, metallic interference screw (IFS), and resorbable IFS fixed group. A tensile tester was used to track the suture wire threaded to the medial meniscus anterior root. The displacement length was recorded after 10 and 20 loading cycles (10–30 N, 100 mm/min cross-head speed). Each specimen was then stretched to failure (50 mm/min cross-head speed), failure modes were recorded, and structural properties (maximum load, linear stiffness, elongation at failure, and elongation at yield) were compared. Fisher’s exact test and one-way analysis of variance were utilized to assess the differences. *Results*: No significant differences in displacement length, upper yield load, maximum load, linear stiffness, elongation at yield, elongation at failure, and frequency of failure mode were observed between the three groups. *Conclusion*: All implants were comparable in terms of fixation strength. Thus, resorbable interference screws may be particularly useful in this technique and does not require implant removal surgery. *Level of evidence*: IV.

## Background

The medial meniscus is secured to the tibia at the posterior root and serves as a secondary knee stabilizer to control tibial rotation and anterior shift [1]. It is responsible for distributing axial loads into hoop stresses during loading [1–5]. Thus, a history of popping during light activities, such as when ascending or descending stairs or walking, characterizes a medial meniscus posterior root tear (MMPRT) [6].

An MMPRT frequently causes knee osteoarthritis caused by a broken meniscal hoop structure [1, 3, 7, 8], with risk factors, including age, female gender, sedentary lifestyle, obesity, and overall varus knee alignment [1–3]. The typical ghost sign or giraffe neck sign is observed in an MMPRT case on magnetic resonance imaging (MRI) [5] and treatment was conservative (non-steroidal anti-inflammatory drugs and exercise therapy) or surgical [9]. Surgical management includes meniscectomy and all-inside suture and fixation using a suture anchor an upcoming technique, MMPRT pull-out repair, which aims to restore the anatomical structure of the meniscus [2–4, 10–12]. In the MMPRT pull-out repair technique, a thread is drawn over the medial meniscus posterior root and pulled out from the anatomical footprint through a bony tunnel on the anteromedial aspect of the tibia and fixed with appropriate tension [7, 13, 14].

In the MMPRT pull-out repair, thread fixation is performed using metal plates and interference screws [15]. However, it frequently damages the medial side soft tissue [11]. Implant selection depends on the operating surgeon’s discretion, and substantial evidence comparing the fixation force of different implants is lacking. Therefore, the present study investigated the strength of different fixation implants for threads pulled out from the anteromedial aspect of the tibia in porcine MMPRT models.

## Methods

### Study design

This biomechanical study included animal experiments conducted at our institution’s biomechanics laboratory under the local Animal Care and Use Committee regulations, with the need for ethical approval waived off by the committee because of the *ex vivo* study design. We used 30 fresh porcine knees (average age: 6 months, weight range: 100–120 kg) obtained from Tokyo Shibaura Zouki, Tokyo, Japan. The specimens were randomly categorized into three groups of 10 specimens each according to the fixation implant used during the MMPRT repair, including (1) double spike plate (DSP; Meira Corp, Nagoya, Aichi, Japan), (2) metallic interference screw (IFS; Cannu-Flex Silk screws, Smith & Nephew Sports Medicine, Andover, MA), and (3) resorbable IFSs MILAGRO (Mitek Sports Medicine, Raynham, MA) fixation groups.

### Surgical procedures

**Figure 1:**
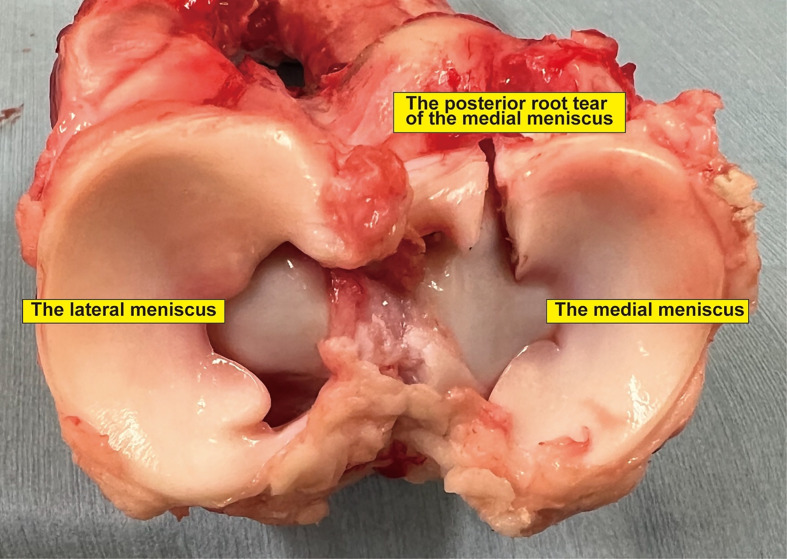
Specimen of a porcine tibia and meniscus. The medial meniscus posterior root was resected to establish a MMPRT model.

**Figure 2:**
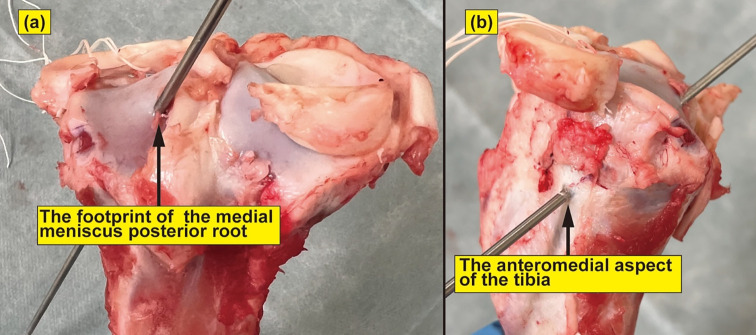
Specimen of porcine tibia viewed from the posterior (a) and medial (b) sides. A 2.4-mm guide pin was inserted from the medial meniscus posterior root footprint toward the anteromedial aspect of the tibia.

**Figure 3:**
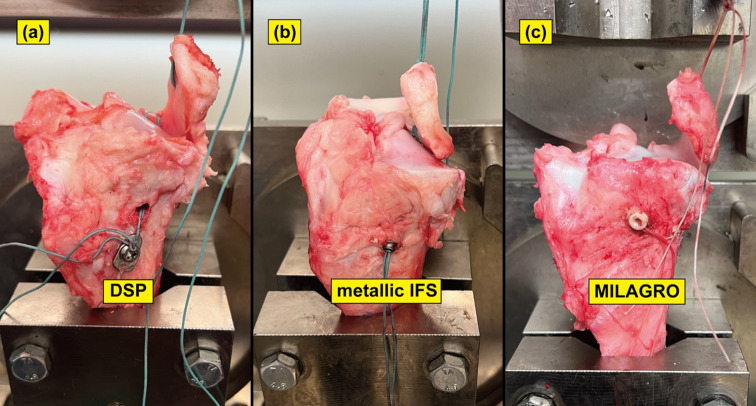
Porcine MMPRT pull-out repair models fixed using three fixation implants, including (a) a DSP, (b) a metallic IFS; CANNU-FLEX SILK SCREWS, Smith & Nephew sports medicine, Andover, MA), and (c) MILAGRO (Mitek Sports Medicine, Raynham, MA). A suture wire was pulled out to the anteromedial aspect of the tibia through a bone tunnel and secured to the implant.

We resected the femur in each specimen and removed all tissues except the tibia and medial meniscus. The medial meniscus posterior root was resected, and an MMPRT model was established according to previous studies [13, 16, 17] (Fig. 1). This model was type 2 in the La Prade classification [18]. A 2.4-mm guide pin was inserted from the medial meniscus posterior root footprint toward the anteromedial aspect of the tibia, and a 4.5-mm drill was utilized to establish a bony tunnel along the guide pin (Figs. 2a and 2b). A suture wire passes through the medial meniscus posterior root, which is pulled out to the anteromedial aspect of the tibia through a bone tunnel. Additionally, fixation was performed using one of the three types of implants: a DSP, a φ 6-mm metallic IFS, or a φ 6-mm MILAGRO with 30 N force of traction (Figs. 3a–3c) [13, 16, 17]. The meniscotibial ligament and the medial meniscus anterior root were resected after fixation, and a suture wire was inserted through the anterior root [13, 16, 17]. We conducted cyclic and rupture tests by tracking this suture wire [13, 16, 17].

### Biomechanical testing of the MMPRT pull-out repair model

**Figure 4:**
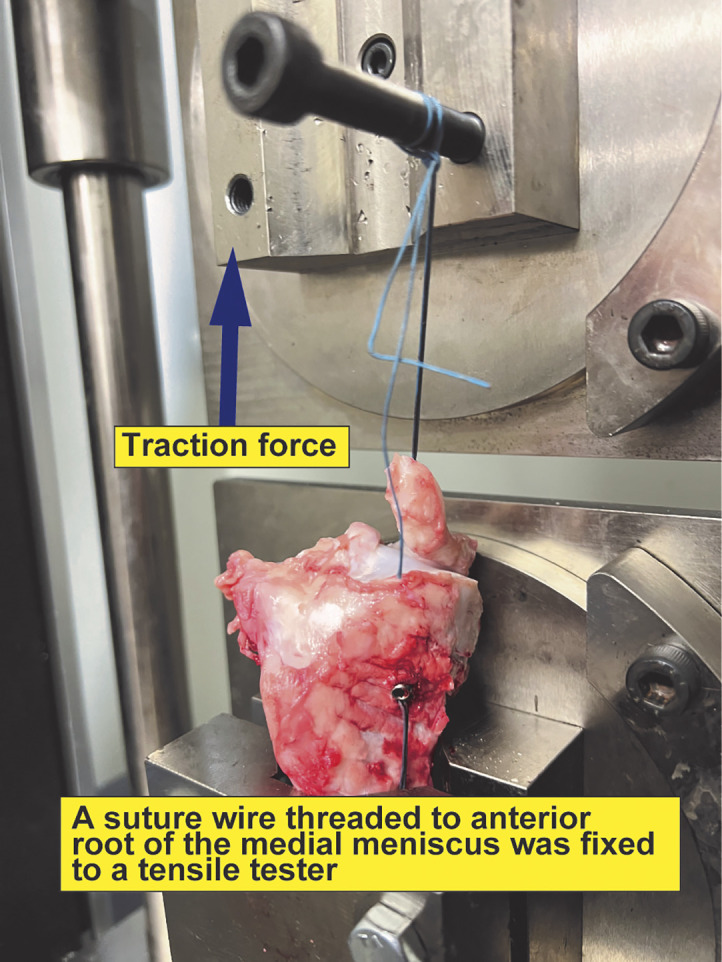
Tensile testing of the porcine MMPRT pull-out repair model using metallic IFS. A suture wire threaded to the medial meniscus anterior root was fixed to a tensile tester. Traction was performed in the arrow’s direction.

All experiments were conducted at room temperature, and the specimens were kept moist with saline solution. The specimens were set to a tensile tester (Tensilon RTG 1250; Orientic, Tokyo, Japan) with specially designed grips, basing all testing protocols on previous studies [13, 14, 19–21]. A suture wire threaded through the medial meniscus anterior root was secured to a tensile tester (Fig. 4). The specimen was preconditioned with a static preload of 5 N for 30 s. Afterward, the elastic modulus of the specimens was assessed by cyclic loading tests at 0.5 Hz, followed by 20 cycles between 10 N and 30 N with a cross-head speed of 100 mm/min [13, 16, 17]. The displacement length was recorded after 10 and 20 tensile loading cycles. Each specimen was then stretched to failure at a cross-head speed of 50 mm/min. Failure modes were recorded, and Tensilon Advanced Controller for Testing (Orientic Co., Ltd., Japan) was used to calculate structural properties (maximum load, linear stiffness, elongation at failure, and elongation at yield).

### Sample size calculation and statistical analysis

An a priori power analysis was performed using G*Power (Heinrich Heine Universität Düsseldorf, Düsseldorf, German) to compute the sample size. Accordingly, a target sample of 30 was identified to achieve an effect size of 0.6 in the mean meniscus dislocation, maximum load, and linear stiffness (*α* < 0.05, power = 0.8).

All data were presented as mean ± standard deviation. Fisher’s exact test was conducted to analyze the failure modes during the rupture test. A one-way analysis of variance was utilized to assess the differences between the three groups. EZR (R Foundation for Statistical Computing, Vienna, Austria) was used for all statistical analyses, with a *p*-value of <0.05 indicating statistical significance.

## Results

### Length change during cyclical loading

No significant differences in the length changes after 10 cyclic tests (mean length change: DSP: 1.3 ± 0.3 mm; metallic IFS: 1.8 ± 0.8 mm; MILAGRO: 2.0 ± 1.3 mm; *p* = 0.22) or 20 cyclic tests (mean length change: DSP: 1.3 ± 0.3 mm; metallic IFS: 1.8 ± 0.8 mm; MILAGRO: 2.0 ± 1.4 mm; *p* = 0.32) were found between the three fixation implant groups (Table 1).

**Table 1:** Results of the length changes during cyclic loading.

Implant	DSP	Metallic IFS	MILAGRO	*p*-value
Displacement after 10 cycles (mm)	1.3 ± 0.3	1.8 ± 0.8	2.0 ± 1.3	0.22
Displacement after 20 cycles (mm)	1.3 ± 0.3	1.8 ± 0.8	2.0 ± 1.4	0.32

DSP: double spike plate (Meira Corp, Nagoya, Aichi, Japan); metallic IFS: interference screw (CANNU-FLEX SILK SCREWS, Smith & Nephew sports medicine, Andover, MA); MILAGRO (Mitek Sports Medicine, Raynham, MA).

### Biomechanical evaluation of the MMPRT pull-out repair models

No significant differences in the mean upper yield load (23.2 ± 9.5 N, 30.5 ± 14.6 N, and 38.3 ± 19.1 N in the DSP, metallic IFS, and MILAGRO groups, respectively; *p* = 0.08), maximum load (98.8 ± 45.3 N, 92.1 ± 32.4 N, and 109.4 ± 35.9 N, respectively; *p* = 0.60), linear stiffness (15.2 ± 7.0 N/mm, 13.9 ± 9.2 N/mm, and 15.9 ± 15.2 N/mm, respectively; *p* = 0.92), elongation at yield (1.9 ± 1.2 mm, 3.0 ± 1.6 mm, and 4.0 ± 2.4 mm, respectively; *p* = 0.05), and elongation at failure (36.0 ± 16.6 mm, 25.8 ± 8.3 mm, and 32.1 ± 13.2 mm, respectively; *p* = 0.23) were observed between the three implant groups (Table 2).

**Table 2:** Results of the traction rupture test.

Implant	DSP	Metallic IFS	MILAGRO	*p*-value
Maximum load (N)	98.8 ± 45.3	92.1 ± 32.4	109.4 ± 35.9	0.60
Stiffness (N/mm)	15.2 ± 7.0	13.9 ± 9.2	15.9 ± 15.2	0.92
Elongation at failure (mm)	36.0 ± 16.6	25.8 ± 8.3	32.1 ± 13.2	0.23
Elongation at yield (mm)	1.9 ± 1.2	2.96 ± 1.6	3.95 ± 2.4	0.05

DSP: double spike plate (Meira Corp, Nagoya, Aichi, Japan); metallic IFS: interference screw (CANNU-FLEX SILK SCREWS, Smith & Nephew sports medicine, Andover, MA).

### Observations of failure mode during the rupture test

**Figure 5:**
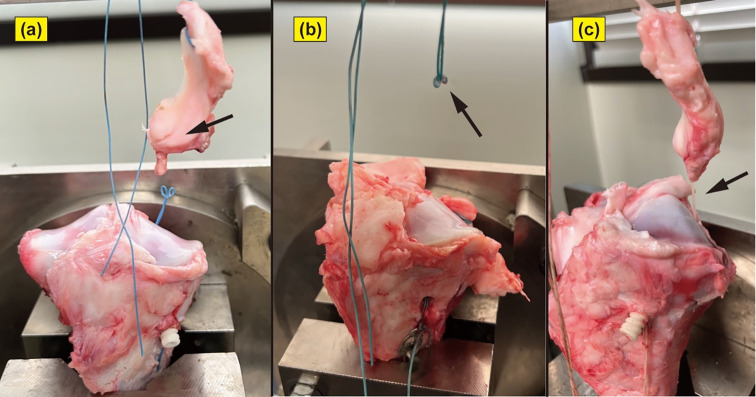
Image illustrating different failure modes in the rupture test of the porcine MMPRT pull-out repair models. (a) Threaded area tears at the anterior horn, (b) posterior horn tears, and (c) thread pull-out from within the bony tunnel.

Threaded area tears at the anterior horn were observed in five specimens in the DSP group and four specimens each in the metallic IFS and MILAGRO groups. Posterior horn tears were observed in 5, 3, and 2 specimens in the DSP, metallic IFS, and MILAGRO groups, respectively. Thread pull-out from within the bony tunnel was observed in 7 specimens, including 0, 3, and 4 in the DSP, metallic IFS, and MILAGRO groups, respectively, with no significant differences between the groups (*p* = 0.31) (Figs. 5a–5c; Table 3).

**Table 3:** Results of the traction failure modes.

Implant	DSP	Metallic IFS	MILAGRO
Tear at the anterior horn (cases)	5	4	4
Tear at the posterior horn (cases)	5	3	2
Pull-out from the bony tunnel (cases)	0	3	4
Total (cases)	10	10	10

DSP: double spike plate (Meira Corp, Nagoya, Aichi, Japan); metallic IFS: interference screw (CANNU-FLEX SILK SCREWS, Smith & Nephew sports medicine, Andover, MA).

## Discussion

The main result of this study indicated no significant difference in displacement, upper yield load, maximum load, linear stiffness, elongation at yield, and elongation at failure for each of the three implants when cyclic and fracture tests were conducted. Furthermore, no significant differences in the frequency of failure modes were observed between implants.

The MMPRT pull-out repair is used for MMPRT to regain the anatomical footprint and reconstruct the meniscal hoop structure. The method of pull-out repair varies from surgeon to surgeon in terms of an artificial ligament, thread, or hamstring tendon being pulled through the medial meniscus posterior root and the selection of fixation implant used [15, 22]. Currently, the optimum choice of fixation implants demonstrated no consensus, some of which include end buttons, DSPs, metallic IFSs, and resorbable IFSs [2, 5, 23–26].

Medial side soft tissue damage occurs during fixation with the implant; thus, this study investigated implants that require as little medial soft tissue invasion and implant removal as possible [11]. A medial open-wedge high tibial osteotomy (MOWHTO), in some cases, is performed in addition to MMPRT pull-out repair to reduce the pressure in the medial knee joint compartment in case of a varus deformity in the lower limb [8]. The bone tunnel may overlap around the medial collateral ligament (MCL) footprint when a bone tunnel is drilled after MOWHTO, followed by plate fixation on the medial aspect of the tibia. Hence, thread fixation using the MMPRT pull-out technique may damage the medial side soft tissue. Our results revealed no significant strength-related differences between the three fixation implants. Furthermore, no studies have reported significant differences in postoperative clinical outcomes with tibial fixation implants in MMPRT pull-out repair. Therefore, MILAGRO is useful in this context as it decreases the damage of medial soft tissue, and the effort required for implant removal. Conversely, the implants used in this study were priced as follows: 21,500 yen for DSPs, 29,600 yen for metallic IFSs, and 46,300 yen for MILAGRO. MILAGRO were approximately 1.5–2 times more expensive than other implants.

This study has several limitations. First, we used a porcine MMPRT model, which is different from the human meniscus. However, pigs are analogous to humans in anatomical structure and physiology, and the use of pigs as a human biological model is considered more suitable than rodents [27].

Second, we could not replicate the *in vivo* knee joint loading scenario on the meniscus after MMPRT pull-out repair because the center of gravity of an 80–100 kg pig is near the foreleg, and the load on the hind leg is lower than that on the foreleg. The load on one hind leg amounts to approximately 160–200 N in quadrupeds [28]. Third, the meniscus anterior root was pulled with a thread, which may have affected the rapture test. Further research is warranted to consider a method that does not load the anterior root using a clamp or a weighting method that is closer to the biomechanics of the body. Fourth, we did not investigate the healing process of MMPRT. Therefore, changes in fixation strength during the healing process are unclear.

## Conclusion

No significant differences in the fixation strength of metal plates, metallic IFSs, and MILAGRO were found when used in an MMPRT pull-out repair technique. Therefore, MILAGRO is particularly useful in this regard because they cause lesser damage to medial soft tissue during fixation and do not require implant removal surgery.

## List of abbreviations

MCL: medial collateral ligament
MMPRT: medial meniscus posterior root tear
MOWHTO: medial open-wedge high tibial osteotomy

## Data Availability

Data and materials of this study are available from the corresponding author on reasonable request.
